# Short-Term Strength Exercise Reduces Hepatic Insulin Resistance in Obese Mice by Reducing PTP1B Content, Regardless of Changes in Body Weight

**DOI:** 10.3390/ijms22126402

**Published:** 2021-06-15

**Authors:** Kellen Cristina da Cruz Rodrigues, Rodrigo Martins Pereira, Guilherme Francisco Peruca, Lucas Wesley Torres Barbosa, Marcella Ramos Sant’Ana, Vitor Rosetto Muñoz, Ana Paula Morelli, Fernando Moreira Simabuco, Adelino Sanchez Ramos da Silva, Dennys Esper Cintra, Eduardo Rochete Ropelle, José Rodrigo Pauli, Leandro Pereira de Moura

**Affiliations:** 1 Exercise Cell Biology Lab, Faculty of Applied Sciences, State University of Campinas, 1300 Pedro Zaccaria Street, Limeira 13484-350, SP, Brazil; kellen.rodrigues.nut@gmail.com (K.C.d.C.R.); rodrigo_mpereira@hotmail.com (R.M.P.); guilhermeperuca1219@gmail.com (G.F.P.); 2 Laboratory of Molecular Biology of Exercise, Faculty of Applied Sciences, University of Campinas, 1300 Pedro Zaccaria Street, Limeira 13484-350, SP, Brazil; torresbarbosa.lucas@gmail.com (L.W.T.B.); vitor.munoz93@gmail.com (V.R.M.); eduardoropelle@gmail.com (E.R.R.); rodrigopaulifca@gmail.com (J.R.P.); 3 Laboratory of Nutritional Genomics, School of Applied Sciences, State University of Campinas, 1300 Pedro Zaccaria Street, Limeira 13484-350, SP, Brazil; marcellarsantana@gmail.com (M.R.S.); dcintra@yahoo.com (D.E.C.); 4 Multidisciplinary Laboratory of Food and Health, Faculty of Applied Sciences (FCA), State University of Campinas (UNICAMP), Limeira 13484-350, SP, Brazil; apm.morelli@hmail.com (A.P.M.); simabuco@gmail.com (F.M.S.); 5 School of Physical Education and Sport of Ribeirão Preto, University of São Paulo, 3900 Bandeirantes Avenue, Ribeirão Preto 14040-907, SP, Brazil; adelinosanchez@usp.br

**Keywords:** strength exercise, obesity, liver, insulin signaling, diabetes, PTP1B, gluconeogenesis

## Abstract

Obesity is closely related to insulin resistance and type 2 diabetes genesis. The liver is a key organ to glucose homeostasis since insulin resistance in this organ increases hepatic glucose production (HGP) and fasting hyperglycemia. The protein-tyrosine phosphatase 1B (PTP1B) may dephosphorylate the IR and IRS, contributing to insulin resistance in this organ. Aerobic exercise is a great strategy to increase insulin action in the liver by reducing the PTP1B content. In contrast, no study has shown the direct effects of strength training on the hepatic metabolism of PTP1B. Therefore, this study aims to investigate the effects of short-term strength exercise (STSE) on hepatic insulin sensitivity and PTP1B content in obese mice, regardless of body weight change. To achieve this goal, obese Swiss mice were submitted to a strength exercise protocol lasting 15 days. The results showed that STSE increased Akt phosphorylation in the liver and enhanced the control of HGP during the pyruvate tolerance test. Furthermore, sedentary obese animals increased PTP1B content and decreased IRS-1/2 tyrosine phosphorylation; however, STSE was able to reverse this scenario. Therefore, we conclude that STSE is an important strategy to improve the hepatic insulin sensitivity and HGP by reducing the PTP1B content in the liver of obese mice, regardless of changes in body weight.

## 1. Introduction

In general, proinflammatory proteins reduce insulin signaling, contributing to the establishment of insulin resistance in different tissues and collaborating with the development of type 2 diabetes *mellitus* (T2DM) in obese subjects [1]. In the hepatocytes, the insulin, after binding to its receptor, initiates a signaling cascade, which will culminate in the phosphorylation of nuclear transcription factor Forkhead Box Protein O1 (FOXO1) promoting its translocation from the cell nucleus to the cytoplasm [2,3]. One of the main functions of FOXO1 in the hepatocyte cell nucleus is to ally itself with Peroxisome Proliferator-Activated Receptor Gamma Coactivator 1 Alpha (PGC1-α), which is a nuclear transcription cofactor, and initiate the transcription of molecules that will collaborate with the gluconeogenesis process in the organ [4]. However, when there is insufficient insulin responsiveness, the FOXO1 phosphorylation is reduced causing transcription of gluconeogenic genes and perpetuating exacerbated gluconeogenesis even in postprandial periods [5]. Thus, identifying proteins that negatively interfere in the hepatic insulin sensitivity and strategies to reduce their content become extremely important to improve the quality of life of obese and diabetic people.

Tyrosine-protein phosphatase non-receptor type 1, also known as protein-tyrosine phosphatase 1B (PTP1B), is a member of the protein tyrosine phosphatase (PTP) family [6]. PTPs are proteins with high potential to negatively regulate insulin transduction since PTP1B binds to and dephosphorylates and inactivates important proteins of the insulin pathway such as Insulin Receptor (IR), as well as Insulin Receptor Substrate (IRS) 1 and 2 [7,8]. The elevation of PTP1B in the liver of obese mice, caused by the administration of Tumor Necrosis Factor-alpha (TNF-α), culminated in severe insulin resistance [9]. On the other hand, when PTP1B was deleted in hepatic tissue, the glycemic homeostasis was improved [10,11]. The authors showed that PTP1B reduces the spread of insulin signaling when PTP1B content is high; it allows greater transcription of gluconeogenic genes. Differently, when it was deleted, this condition was reverted. In this sense, it is evident that PTP1B is an important molecule responsible for glycemic homeostasis, thus strategies that are effective in reducing its content might be relevant to improving insulin sensitivity in obese and diabetic individuals [8]. Since the use of medications can bring unwanted side effects, non-pharmacological strategies that may reduce PTP1B content are of paramount importance.

Regular physical exercise is well known to reduce inflammation resulting from obesity and, consequently insulin resistance in both peripheral and central tissues [12,13,14]. In 2013, Moura and colleagues showed that aged animals presented an elevation of hepatic PTP1B levels and after only one bout of aerobic physical exercise, it was possible to observe PTP1B reduction in this tissue and consequent reduction in the hepatic glucose production (HGP) [15]. In 2015, Passos and co-authors observed that obese animals present increased PTP1B and, after being submitted to a chronic aerobic exercise protocol (8 weeks), presented a reduction in PTP1B and an improvement in glycemic homeostasis [16]. On the other hand, researches involving strength physical exercise, and insulin sensitivity are still little explored in the literature. Recently, we showed that a short period of strength physical exercise was effective in reducing insulin resistance and fat accumulation in the liver of obese mice [17]. However, it is not known whether these findings are linked to PTP1B metabolism. We hypothesize that diet-induced obesity would increase the PTP1B content in the liver of obese animals and impair hepatic insulin sensitivity due to the reduction of IRS-1/2 phosphorylation. On the other hand, we also hypothesize that the short-term strength exercise would reverse this scenario, reducing levels of PTP1B, allowing increased phosphorylation of IRS-1/2 in Y612 residue, even without promoting body weight change. In this sense, since there are no studies showing the direct effects of strength training on the metabolism of PTP1B, this study aimed to investigate the effects of STSE on hepatic insulin sensitivity and whether a short period of strength physical exercise can decrease PTP1B content and increased tyrosine phosphorylation of IRS-1, allowing increased activity of Akt, which culminate in lower fasting glycemia and better HGP control, regardless of changes in body weight.

## 2. Results

### 2.1. Short-Term Strength Exercise Ameliorates Glucose Sensitivity without Reducing Body Weight and Fat Depots

**Figure 1:**
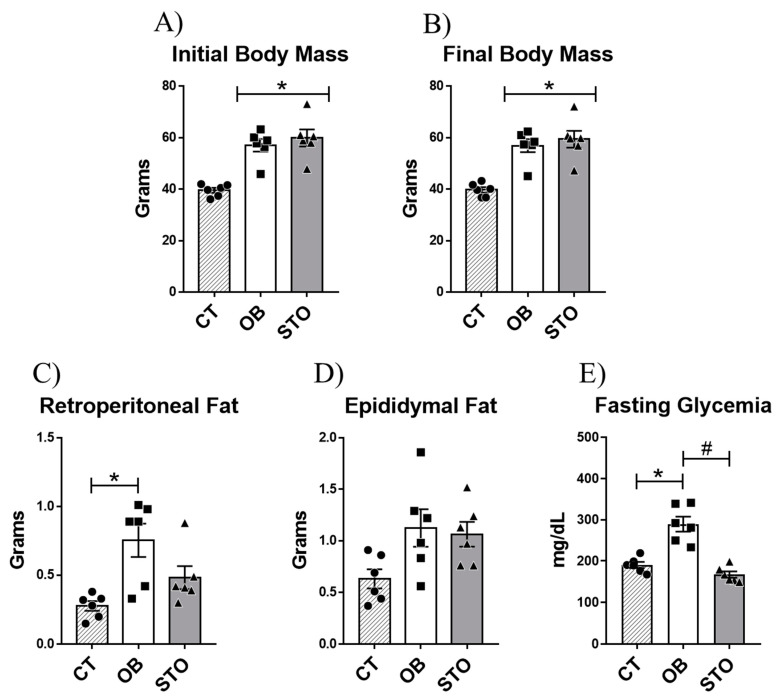
Physiological parameters. Initial body mass: body weight of the animals after diet-induced obesity protocol and before starting the exercise protocol (**A**), Final body mass: body weight of the animals after short-term strength exercise protocol (15 exercise sessions) and before euthanasia (**B**), Retroperitoneal fat (**C**), Epididymal fat (**D**), and Fasting glycemia (**E**). *n* = 6 per group. CT = Control group; OB = Obese Sedentary Group and STO = Strength Training Obese. * *p* < 0.05 vs. CT; # *p* < 0.05 vs. OB (*n* = 6 per group).

To evaluate the effects of STSE in obese mice the first step of this study was to induce obesity using HFD for 14 weeks. After that, the obese animals were divided into sedentary and exercised mice. The exercised animals were submitted to 15 sessions of strength exercise which consisted of 20 climbing series with an overload of 70% of the maximum voluntary carrying capacity (MVCC) and with a rest interval of 60–90 s between sets. After the experimental period, the results demonstrate that the diet-induced obesity protocol was effective in increasing body weight gain and impairing glucose sensitivity (Figure 1A,B,E). Besides, 15 sessions of strength training were able to revert the hyperglycemia caused by obesity regardless of body weight chance or epididymal fat and retroperitoneal fat reduction (Figure 1A–E).

### 2.2. Short-Term Strength Exercise Improves Insulin Sensitivity and Reduces Hepatic Glucose Production in Obese Mice

**Figure 2:**
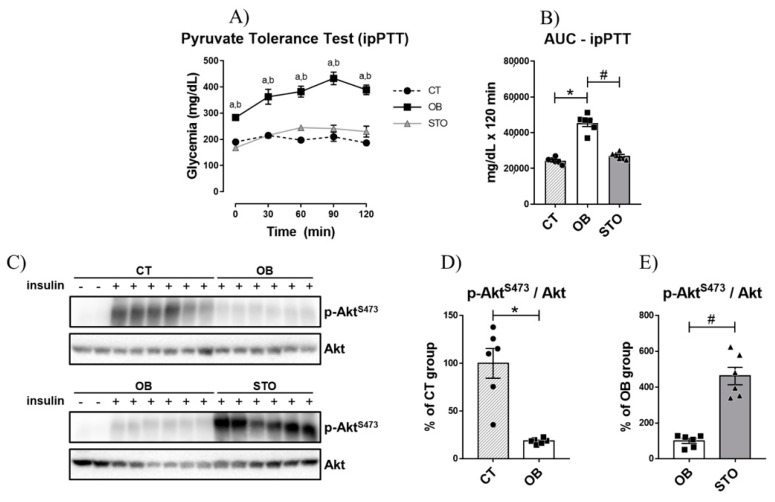
Hepatic Glucose Production and Hepatic Insulin Signaling. Glycemic curve during Intraperitoneal Pyruvate Tolerance Test (ipPTT) (**A**); Area Under the Curve (AUC) during ipPTT (**B**), Bands of phospho-Akt^S473^ and total Akt (**C**), Quantification of p-Akt^S473^/Akt of CT and OB groups (**D**); Quantification of p-Akt^S473^/Akt of OB and STO groups (**E**). *n* = 6 per group. CT = Control group; OB = Obese Sedentary Group and STO = Strength Training Obese. In (**A**): a = *p* < 0.05 for CT vs. OB; b = *p* < 0.05 for OB vs. STO. In (**B**,**D**,**E**): * *p* < 0.05 vs. CT; # *p* < 0.05 vs. OB. CT: *n* = 8 (2 saline injection + 6 insulin injection)—OB: *n* = 8 (2 saline injection + 6 insulin injection)—STO *n* = 6 (all insulin injection). The statistical analysis was performed with insulin injected mice.

Once it was observed that obesity impaired fasting glycemia, it was sought to investigate whether hepatic glucose production was affected by obesity. The results of the intraperitoneal pyruvate tolerance test (ipPTT) demonstrated that obese sedentary animals presented increased hepatic glucose production, as the blood glucose was higher at all time points of the test when compared to the control (Figure 2A), culminating in a greater area under the curve (AUC) during the ipPTT (Figure 2B). In contrast, short-term strength exercise ameliorates HGP control, reducing blood glucose during ipPTT as well as the AUC of the test (Figure 2A,B). Also, obesity impaired insulin signaling in the liver of mice (Figure 2C,D); however, exercised mice restored Akt phosphorylation, demonstrating an improvement in hepatic insulin signaling (Figure 2C,E).

### 2.3. Strength Exercise Reduces PTP1B Content in the Liver of Obese Mice

**Figure 3:**
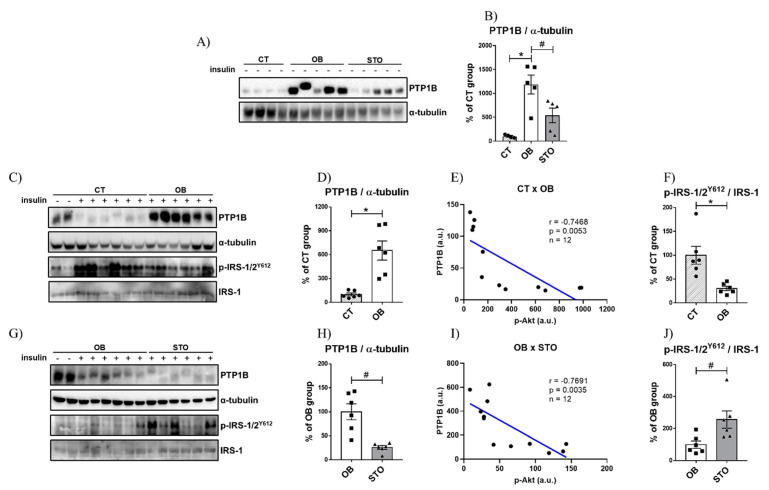
PTP1B content is increased in obese mice and STO downregulates PTP1B content. Bands of basal PTP1B, β-actin p-IRS-1/2 (Y612) and total IRS-1 of CT, OB, and STO groups (**A**), Quantification of basal PTP1B normalized with α-tubulin of CT, OB, and STO groups (**B**), Bands of PTP1B and α-tubulin of CT and OB groups (**C**), Quantification of PTP1B normalized with α-tubulin of CT and OB groups (**D**), Correlation between the protein content of PTP1B and p-Akt of CT and OB groups (**E**), Quantification of p-IRS-1/2 (Y612) normalized with total IRS-1 of CT and OB groups (**F**), Bands of PTP1B and α-tubulin of OB and STO groups (**G**), Quantification of PTP1B normalized with α-tubulin of OB and STO groups (**H**), Correlation between the protein content of PTP1B and p-Akt of CT and OB groups (**I**), Quantification of p-IRS-1/2 (Y612) normalized with total IRS-1 of OB and STO groups (**J**). CT = Control group; OB = Obese Sedentary Group and STO = Strength Training Obese. * *p* < 0.05 vs. CT; # *p* < 0.05 vs. OB. Panels A–B: *n* = 4–6 animals per group—all saline-injected animals. Panels C–H: CT: *n* = 8 (2 saline injection + 6 insulin injection)—OB: *n* = 8 (2 saline injection + 6 insulin injection)—STO *n* = 6 (all insulin injection). The statistical analysis in panels C–H was performed with insulin-injected mice.

The next step of this study was to evaluate the effects of two interventions—obesity and exercise—in the basal level of PTP1B. To achieve this aim, we measured the protein content of PTP1B in control, sedentary obese, and strength training obese animals. The results in Figure 3A,B show that diet-induced obesity (DIO) robustly increased the basal PTP1B content in sedentary animals, however, STSE reduced the basal PTP1B levels. Subsequently, our objective was to investigate the effects of obesity and physical exercise on obese animals stimulated with insulin. When comparing the PTP1B levels in CT and OB mice stimulated with insulin, it is possible to observe that PTP1B is increased in OB mice (Figure 3C,D), similarly as observed in the PTP1B basal levels (without insulin stimulation). Consequently, it was possible to observe that obese animals showed a reduction in p-IRS-1/2^Y612^ levels after insulin stimulus (Figure 3C,F). Furthermore, by evaluating the effects of physical exercise in insulin-stimulated animals, the STSE was able to reduce the PTP1B content when compared to sedentary obese animals (Figure 3G,H). As a consequence of the reduction in PTP1B caused by exercise, the p-IRS-1/2^Y612^ levels were elevated (Figure 3G,J). Moreover, to assess if there is a correlation between PTP1B and insulin signaling in this study, the protein levels of PTP1B were correlated with p-Akt content. The protein content of PTP1B was negatively correlated with p-Akt in both comparisons CT × OB (Figure 3E) and OB × STO (Figure 3I).

**Figure 4:**
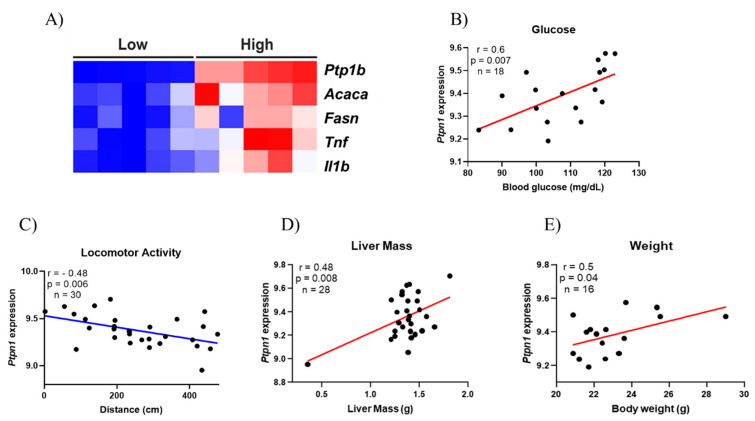
Bioinformatics analysis. Heatmap illustrating the lower and higher expression of Protein-Tyrosine Phosphatase 1B (PTP1B) and the influence in lipogenic genes [Acetyl-CoA Carboxylase (ACC) and Fatty Acid Synthase (FAS)] and inflammatory genes [Tumor Necrosis Factor (TNF) and interleukin 1β (IL1β)] on human liver tissue. The figure illustrates clusters between the groups Low and High showing that they are directly related (**A**); Correlation between PTP1B gene expression and Blood Glucose levels (*n* = 18) (**B**); Correlation between PTP1B gene expression and locomotor activity (*n* = 30) (**C**); Correlation between PTP1B gene expression and liver mass (*n* = 28) (**D**); Correlation between PTP1B gene expression and body weight (*n* = 16) (**E**).

Furthermore, a publicly accessible dataset from human liver samples was used to evaluate the expression of PTP1B, lipogenic, and inflammatory genes. It was observed that when there is a high amount of *Ptp1b* mRNA, the expression of *Acaca*, *Fasn*, *Tnf*, and *Il1b* is also increased (Figure 4A). Moreover, using obese mice liver dataset, it was evaluated if there could be a correlation between *Ptp1b* levels in the liver of those mice with the following phenotypes: Blood glucose, locomotor activity, liver mass, and body weight (Figure 4B–E). Almost all phenotypes evaluated showed a positive correlation with PTP1B, except for the locomotor activity, highlighting our hypothesis that physical exercise might be an interesting strategy to reduce PTP1B content and, consequently, ameliorating hepatic glycemic control.

## 3. Discussion

Obesity is a major risk factor to the development of hepatic insulin resistance culminating in fasting hyperglycemia and T2DM genesis [18,19]. The liver is a key organ for controlling glucose homeostasis since it regulates gluconeogenesis and glycogenolysis, controlling the hepatic glucose release [20]. Also, the hepatic insulin resistance contributes to uncontrolled HGP, which in turn, is majorly responsible for hyperglycemia in T2DM patients [21]. Therefore, it is important to find new strategies to increase hepatic insulin sensitivity and to reduce hepatic gluconeogenesis. Also, studies with animals [22,23] and humans [24] demonstrated that physical exercise is a great strategy to improve hepatic insulin sensitivity. Zhang and colleagues evaluated the effects of acute and chronic aerobic exercise on hepatic insulin sensitivity of diet-induced obese rats and observed that both protocols were able to increase Akt phosphorylation and insulin release [23].

Moreover, the Otsuka Long-Evans Tokushima Fatty (OLETF) rats were another model of obesity and type 2 diabetes used to evaluate the role of aerobic exercise on hepatic insulin sensitivity [22]. The authors submitted OLEFT animals to a voluntary running wheel for 20 weeks and observed that exercised animals increased the phosphorylation of Akt in both Threonine 308 and Serine 473 residues [22]. Furthermore, Malin and colleagues evaluated the effects of 12 weeks of aerobic treadmill walking in 20 older adults, and they observed that exercise was able to reduce hepatic insulin resistance, which was assessed using the euglycemic–hyperinsulinemic clamp [24]. On the other hand, there is a lack of studies on the role of strength exercise in hepatic insulin sensitivity in the literature. In this study, we showed that even without reducing body weight, a short-term strength exercise protocol improved hepatic insulin signaling since STSE animals decreased PTP1B content and increased phosphorylation of IRS-1, allowing increased activity of Akt, which culminated in lower fasting glycemia and better HGP control.

An interesting target to improve hepatic insulin signaling is PTP1B since this phosphatase can dephosphorylate the insulin receptor and its substrates and block insulin signaling [25]. Panzhinskiy and colleagues observed that PTP1B whole-body knockout mice attenuated the harmful effects of 20 weeks of HFD, such as body weight gain, adiposity, glucose intolerance, and hepatic steatosis when compared with wild-type C57BL/6J mice that were also fed an HFD, suggesting that PTP1B is an important protein in obesity development [26]. Studies with liver-specific knockout mice have shown an overall glucose homeostasis improvement, including enhanced hepatic insulin signaling and increased suppression of hepatic glucose production in insulin-resistant and high fat diet-induced obesity models [11,27]. Moreover, there is evidence in the literature showing that Akt might impair PTP1B function as a positive feedback mechanism of insulin action since PTP1B could impair the upstream sites to inhibit insulin signaling [28]. Using the cell culture model, Ravichandran and colleagues showed that Akt can phosphorylate PTP1B at Serine 50 after insulin stimulation, and they also observe that mutations at Serine 50 affect the ability of PTP1B to dephosphorylate insulin receptor (IR) and IRS [28]. As observed in our data, STSE increased Akt phosphorylation which can have reduced PTP1B activity in the cellular cytosol, culminating in increased IRS-1/2 tyrosine phosphorylation and better hepatic insulin signaling. Interestingly, our data showed a negative correlation between the PTP1B protein content and Akt phosphorylation, when comparing CT vs. OB and OB vs. STO groups, which indicates that those proteins are related to each other.

Physical exercise seems to be a great strategy to reduce PTP1B content and improve insulin signaling. Moura and colleagues evaluated the effects of acute aerobic exercise on the liver content of PTP1B of aged mice and observed that two bouts of swimming exercise were able to reverse the increased content of PTP1B due to the aging process. Moreover, aged-exercised mice enhanced insulin signaling and reduced the gluconeogenesis pathway, suggesting that PTP1B content reduction mediated by physical exercise might be related to better glucose homeostasis [15]. In contrast, little is known about the effects of strength exercise on hepatic insulin sensitivity and HGP. Botezelli and colleagues compared the effects of chronic (8 weeks) aerobic, strength, and combined exercise in fructose-fed rats, and observed that the strength exercise protocol (which consists of series of jumps in tanks of water) showed improvements in glucose homeostasis, insulin sensitivity and the content of lipids in hepatic tissue, which highlights the significance of investigating this type of exercise [29]. Recently our research group showed that short-term strength exercise reduced the HGP and improved insulin signaling in the liver [17]; however, there is a gap in the literature about the role of this type of exercise on PTP1B association with proteins of the insulin pathway. This is the first study showing the efficiency of strength exercise to reduce PTP1B content in obese mice without body weight interference, and it seems to be an important non-pharmacological option to treat the complications of obesity and T2DM. Moreover, it is important to highlight the isolated effect of STSE impacting the PTP1B levels, since the basal levels of PTP1B in non-insulin stimulated animals were also reduced in exercised mice when compared with sedentary obese animals.

**Figure 5:**
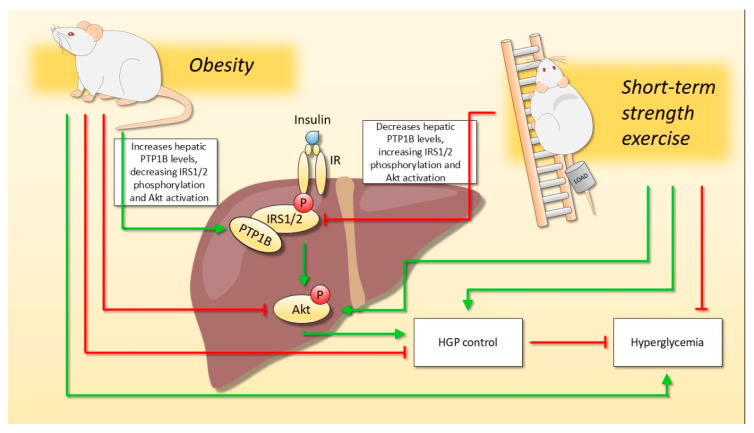
The effect of short-term strength exercise on hepatic glucose metabolism, regardless of body weight change. Diet-induced obesity impaired hepatic insulin signaling due to increased PTP1B content, which results in hyperglycemia and impaired control of hepatic glucose production (HGP). On the other hand, short-term strength exercise was able to reverse the harmful effects of obesity since exercised animals reduced the PTP1B content. Moreover, even though the exercised animals did not reduce body weight, the hepatic insulin signaling was improved, and they demonstrated better HGP control and reduced fasting glycemia.

In conclusion, short-term strength exercise can improve hepatic insulin signaling, increasing IRS-1/2 tyrosine phosphorylation, and Akt activation, as well as lowering hepatic glucose production and fasting glycemia. Also, we showed that hepatic insulin sensitivity was increased due to a reduction in PTP1B content, as observed in exercised animals, reducing IRS-1/2 tyrosine dephosphorylation (Figure 5). It is important to highlight that these benefits were found regardless of body weight reduction in obese mice. Therefore, strength exercise may be considered an important strategy to prevent and treat the side effects of obesity and associated diseases such as T2DM, improving metabolic health even in the earlier stages of the treatment.

## 4. Materials and Methods

### 4.1. Animals and Diet

Male Swiss mice at eight weeks old were used in the present study. The animals were from the Unicamp Central Animal Facility (CEMIB), and all animals experiments were previously approved by the Ethics Committee on Animal Use (CEUA) of Biological Sciences (UNICAMP-Campinas-SP, number 4406-1) and carried out according to the Brazilian legislation on the scientific use of animals (Law No. 11.794, of 8 October 2008). The animals arrived at four-week-old and were maintained in individual polyethylene cages with an enriched environment, and with inside light, noise, humidity, and temperature control, as we previously described [17]. Water and conventional food were offered ad libitum.

Initially, the animals were divided into two groups: the Control Lean (CT) group (*n* = 8) was fed a chow diet, and the Obesity group was fed a high-fat diet (HFD). The diet-induced obesity protocol lasted 14 weeks, and after that, the animals of the obese group were equally redistributed, considering their body weight and fasting glycemia, into two groups: (a) Sedentary Obese (OB) (*n* = 8), which remained sedentary throughout the experiment and (b) Strength Training Obese (STO) (*n* = 6), which was submitted to a short-term strength training. The high-fat diet was prepared according to the American Institute of Nutrition (AIN-93G) guidelines [30], modified to contain 35% of fat (4% soy oil and 31% of lard) [31]. Moreover, it is important to highlight that all experiments were repeated to evaluate the basal levels of PTP1B, therefore, the diet-induced obesity protocol and the short-term strength training were performed twice. The number of animals used in the second experiment was: CT—*n* = 4, OB—*n* = 5, and STO—*n* = 5.

### 4.2. Experimental Design and Exercise Protocol

The short-term strength training was performed on a ladder with a 1.5 cm distance between the steps and 70 cm high (AVS projects, São Carlos, Brazil), and the mice carried the load apparatus fixed with adhesive tape across the length of their tail. The load apparatus was a conical plastic tube with around 7.5 cm of height and 2.5 cm of diameter.

The first step was the adaptation to the apparatus that lasted five consecutive days. On the first day, the animals were placed in a chamber at the top of the ladder for 60 s, with the loading apparatus empty attached on its tail. The animals were progressively placed away from the chamber. For the first climbing attempt, the animal was placed on the ladder at 15 cm from the entrance of the chamber. For the second attempt, the animal was placed 25 cm away from the chamber. For the third attempt onwards, the animal was positioned at the base of the ladder, 70 cm away from the chamber. The attempts started from the base of the ladder and continued until the animal reached the chamber three times without the need for any stimulus.

Then, 48 h after the last day of adaptation, the animals were submitted to the maximal voluntary carrying capacity (MVCC) test to determine the maximum load with which each animal could climb the entire length of the ladder. The MVCC started with an overload corresponding to 75% of the animal’s body weight, and an incremental load of 5 g was added at each further attempt to climb until the animal could no longer complete the entire course. At the end of each successful attempt, the animal rested in an individual cage for 5 min until the next attempt. The heaviest overload in which the animal performed a successful climb was considered the MVCC and this value was used to prescribe the individual loads in the experiment.

**Figure 6:**
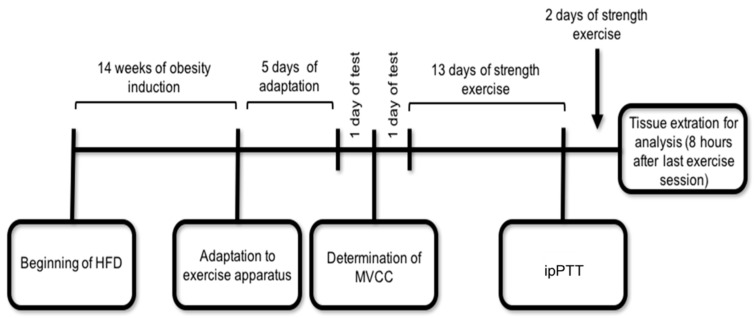
Schematic representation of the experimental design. The ipPTT and tissue extraction were performed 8 h after the exercise session and 8-h fasting. HFD = High Fat Diet; MVCC = Maximum Voluntary Carrying Capacity; ipPTT = intraperitoneal Pyruvate Tolerance Test.

The strength exercise protocol began 48 h after the MVCC determination. The exercise sessions consisted of 20 climbing series with an overload of 70% of the MVCC and with a rest interval of 60–90 s between sets. The animals were exercised for five consecutive days per week, followed by two days of rest, until they completed 13 sessions of physical exercise. Subsequently, mice were submitted to the pyruvate tolerance test. After 24 h, the animals performed two more sessions of exercise, totaling 15 sessions, as demonstrated in Figure 6.

### 4.3. Intraperitoneal Pyruvate Tolerance Test (ipPTT)

To estimate the hepatic glucose production (HGP) control, an ipPTT was performed after 13 exercise sessions. The ipPTT protocol consists of intraperitoneal injection of 2.0 g of pyruvate/kg body weight after 8 h fasting and 8 h after the last exercise session. The blood samples were collected at 0, 30, 60, 90, and 120 min from the tail of the animal for blood glucose determination. Point 0 was collected before pyruvate (Êxodo Científica, Sumaré, SP, Brazil) injection. The results were evaluated by determining the areas under the blood glucose curves (AUC) during the test by the trapezoidal method [32], using Microsoft Excel (Version 2013).

### 4.4. Fasting Glycemia Assay

Eight hours after the 15th session of STSE, and 8 h fasting, the blood was collected from the tail of the animals to determine the fasting glycemia. The blood glucose was determined using a glucometer (Accu-Chek; Roche Diagnostics, Indianapolis, IN, USA).

### 4.5. Tissue Extraction and Immunoblotting Analysis

After 8 h fasting and 8 h after the last exercise session, before receiving saline or insulin, all animals were anesthetized via i.p. by the injection of chloral hydrate of ketamine (50 mg/kg, Parke-Davis, Ann Arbor, MI, USA) and xylazine (20 mg/kg, Rompun, Bayer, Leverkusen). After the verification and assurance of the corneal reflexes, mice were injected via i.p. with human insulin (8 U/kg body wt Humulin-R; Lilly, Indianapolis, IN, USA) or saline. The first time that the experiment was performed 2 animals from the CT group and 2 animals from the OB group received saline and 6 animals from each group (CT, OB, and STO) received insulin. On the other hand, in the second time that this experiment was conducted all animals received only the saline injection (CT—*n* = 4, OB—*n* = 5, and STO—*n* = 5) because this study aimed to analyze the basal levels of PTP1B. After 10 min, the liver was rapidly removed and snap-frozen in liquid nitrogen and stored at −80 °C until analysis and epididymal and retroperitoneal adipose tissue (right side) were removed and weighed. The liver was homogenized in an extraction buffer [1% Triton-X 100, 100 mM Tris (pH 7.4), 100 mM sodium pyrophosphate, 100 mM sodium fluoride, 10 mM EDTA, 10 mM sodium vanadate, 2 mM PMSF and 0.1 mg of aprotinin/mL] at 4 °C with a TissueLyser II (QIAGEN^®^) operated at maximum speed for 120 s. The lysates were centrifuged (Eppendorf 5804R) at 12.851 g at 4 °C for 15 min to remove insoluble material, and the supernatant was used for the assay. The protein content was determined by the bicinchoninic acid method [33]. The samples containing 60 μg of total protein were applied to a polyacrylamide gel for separation by SDS-PAGE and transferred to nitrocellulose membranes. The membranes were blocked with 5% dry milk at room temperature for 1 h and incubated with primary antibodies against the protein of interest. After that, a specific secondary antibody was used. The specific bands were labeled by chemiluminescence and visualization was performed by a photo documentation system in G: box (Syngene). The bands were quantified using the software UN-SCAN-IT gel 6.1. The primary antibodies used were: anti-Phospho-Akt S473 (4060), anti-Akt (4685) and anti- α-Tubulin (2144) from Cell Signaling Technology^®^ (Beverly, MA, USA) and anti-PTP1B (sc14021), anti-IRS-1 (sc559), and Phospho-IRS-1/2 Y612 (sc17195) from Santa Cruz Biotechnology^®^ (Santa Cruz, CA, USA). The secondary antibody used was the Anti-rabbit IgG, from Cell Signaling Technology^®^ (Beverly, MA, USA).

### 4.6. Bioinformatics Analysis

A heatmap was made using the data collected in Gene Network (http://www.genenetwork.org/, accessed on 2 October 2019) using the GTExv5 Human Liver RefSeq (Sep15) RPKM log2 data set of mRNA expression [34]. Correlations were made using obese mice liver dataset “EPFL/LISP BXD HFD Liver Affy Mouse Gene 1.0 (Aug18) RMA”, also available on GeneNetwork. Phenotypes were only included when data were collected at the same time, excluding time-courses and also, excluding data when mice had interventions such as stress, drug injections, or exposure to different types of environments. Data were normalized and distributed according to the z-score distribution, using R Project studio (Version 1.2.5033). More details of the steps of the bioinformatic analysis can be found in the Supplementary Material.

### 4.7. Statistical Analysis

All results were presented as the mean ± standard error of the mean (SEM). The Gaussian distribution of the data was assessed using a Shapiro–Wilk test and analyzed by Student’s *t*-test for parametric data to compare two groups when it was necessary. We used the one-way Analysis of Variance (ANOVA) test followed by Bonferroni’s post-hoc test to compare more than two groups. Two-way ANOVA (with repeated measures when appropriate), with Bonferroni’s correction for multiple comparisons, was used to analyze each point of ipPTT. The level of statistical significance used was *p* < 0.05. The construction of the graphics and the statistical analysis was performed using GraphPad Prism 7.00.

## Data Availability

Not applicable.

## Supplementary Materials

Supplementary materials can be found at https://www.mdpi.com/article/10.3390/ijms22126402/s1.

## Abbreviations

T2DM	Type 2 Diabetes *Mellitus*
FOXO1	Forkhead Box Protein O1
PGC-1α	Peroxisome Proliferator-Activated Receptor Gamma Coactivator 1 Alpha
PTP1B	Protein-Tyrosine Phosphatase 1B
PTP	Protein Tyrosine Phosphatase
IR	Insulin Receptor
IRS	Insulin Receptor Substrate
TNF-α	Tumor Necrosis Factor-alpha
HGP	Hepatic Glucose Production
STSE	Short-term strength exercise
ipPTT	intraperitoneal Pyruvate Tolerance Test
AUC	Area Under the Curve
ACC	Acetyl-CoA Carboxylase
FAS	Fatty Acid Synthase
IL1β	Interleukin 1β
OLETF	Otsuka Long-Evans Tokushima Fatty
CEMIB	Unicamp Central Animal Facility
CEUA	Ethics Committee on Animal Use
CT	Control Lean
HFD	High-fat diet
OB	Sedentary Obese
STO	Strength Training Obese
AIN-93	American Institute of Nutrition
MVCC	Maximal Voluntary Carrying Capacity
SEM	Standard error of the mean
ANOVA	Analysis of Variance
