# Supplementary material for: Short-Term Strength Exercise Reduces Hepatic Insulin Resistance in Obese Mice by Reducing PTP1B Content, Regardless of Changes in Body Weight
Source: Int J Mol Sci. 2021 Jun 15;22(12):6402. doi: 10.3390/ijms22126402 (PMC8232771; doi:10.3390/ijms22126402)

Short-term strength exercise reduces hepatic insulin resistance in obese mice by reducing PTP1B content, regardless of changes in body weight

Figure 1A: Initial Body Mass

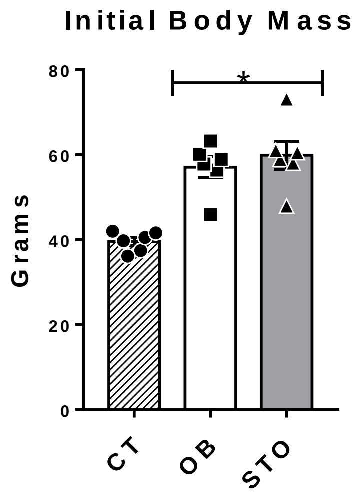

Group A	Group B	Group C
CT	OB	STO
Y	Y	Y
37.4	45.9	47.9
36.1	56.4	58.9
40.5	57.8	61.0
39.7	60.1	58.0
41.6	58.9	73.1
42.0	63.2	60.5

Bonferroni's multiple comparisons test	Mean Diff.	95.00% CI of diff.	Significant?	Summary	Adjusted P Value
CT vs. OB	-17.5	-26.73 to -8.273	Yes	***	0.0004
CT vs. STO	-20.35	-29.58 to -11.12	Yes	****	<0.0001
OB vs. STO	-2.85	-12.08 to 6.377	No	ns	>0.9999

Figure 1B: Final Body Mass

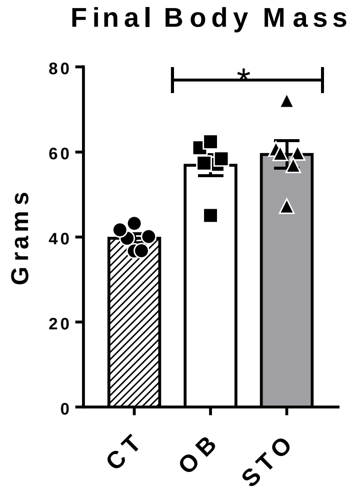

CT	OB	STO
Y	Y	Y
36.7	45.1	47.3
36.8	57.1	59.8
40.1	57.4	60.7
39.7	61.0	56.9
41.7	58.4	72.1
43.2	62.4	59.7

Bonferroni's multiple comparisons test	Mean Diff.	95.00% CI of diff.	Significant?	Summary	Adjusted P Value
CT vs. OB	-17.2	-26.52 to -7.881	Yes	***	0.0005
CT vs. STO	-19.72	-29.04 to -10.4	Yes	***	0.0001
OB vs. STO	-2.517	-11.84 to 6.802	No	ns	>0.9999

Figure 1C: Retroperitoneal Fat

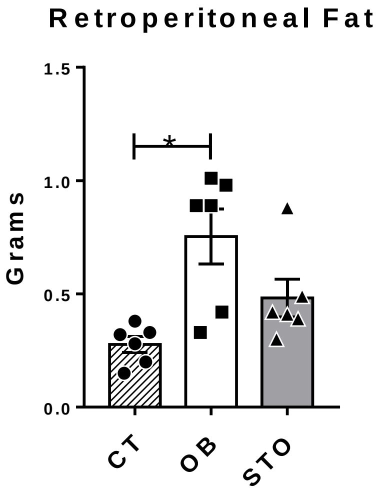

Group A	Group B	Group C
CT	OB	STO
Y	Y	Y
0.15	0.33	0.42
0.20	0.89	0.88
0.33	0.98	0.41
0.38	1.01	0.30
0.32	0.89	0.49
0.28	0.42	0.39

Bonferroni's multiple comparisons test	Mean Diff.	95.00% CI of diff.	Significant?	Summary	Adjusted P Value
CT vs. OB	-0.4767	-0.8106 to -0.1428	Yes	**	0.0048
CT vs. STO	-0.205	-0.5389 to 0.1289	No	ns	0.3567
OB vs. STO	0.2717	-0.06222 to 0.6056	No	ns	0.1338

Figure 1D: Epididymal Fat

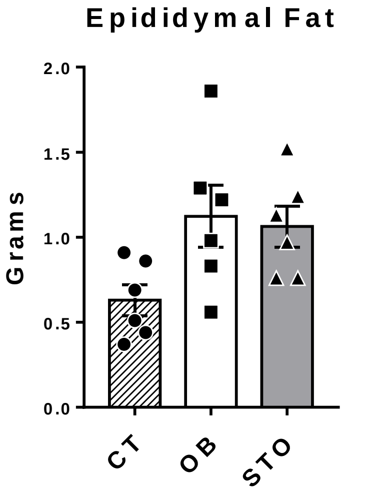

Group A	Group B	Group C
CT	OB	STO
Y	Y	Y
0.44	0.83	1.24
0.37	0.56	1.13
0.91	1.22	0.76
0.86	1.86	1.52
0.69	1.29	0.76
0.51	0.98	0.97

Bonferroni's multiple comparisons test	Mean Diff.	95.00% CI of diff.	Significant?	Summary	Adjusted P Value
CT vs. OB	-0.4933	-1.016 to 0.02926	No	ns	0.0675
CT vs. STO	-0.4333	-0.9559 to 0.08926	No	ns	0.1235
OB vs. STO	0.06	-0.4626 to 0.5826	No	ns	>0.9999

Figure 1E: Fasting Glycemia

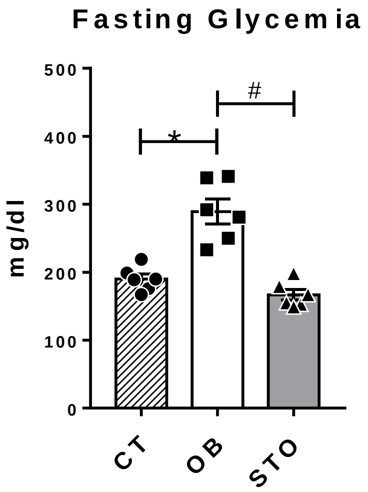

Group A	Group B	Group C
CT	OB	STO
Y	Y	Y
176	250	153
190	233	167
199	341	198
167	339	179
219	281	155
189	292	149

Bonferroni's multiple comparisons test	Mean Diff.	95.00% CI of diff.	Significant?	Summary	Adjusted P Value
CT vs. OB	-99.33	-145.7 to -52.94	Yes	***	0.0001
CT vs. STO	23.17	-23.23 to 69.56	No	ns	0.5958
OB vs. STO	122.5	76.1 to 168.9	Yes	****	<0.0001

Figure 2A: Pyruvate Tolerance Test

Table format: XY		X	Group A							Group B						Group C					
		X Title	CT							OB						STO					
		X	A:Y1	A:Y2	A:Y3	A:Y4	A:Y5	A:Y6	A:Y7	B:Y1	B:Y2	B:Y3	B:Y4	B:Y5	B:Y6	C:Y1	C:Y2	C:Y3	C:Y4	C:Y5	C:Y6
1	0	0	184	194	210	176	198	177		258	291	304	261	321	265	170	182	162	172	159	162
2	30	30	209	218	230	201	210	222		230	431	378	351	398	387	220	240	199	215	205	214
3	60	60	186	199	198	184	201	214		310	398	345	369	421	451	241	261	246	230	258	231
4	90	90	210	201	198	159	289	203		398	501	370	510	419	399	287	262	231	221	246	199
5	120	120	194	140	193	185	201	205		331	463	377	404	362	397	199	298	187	199	293	201

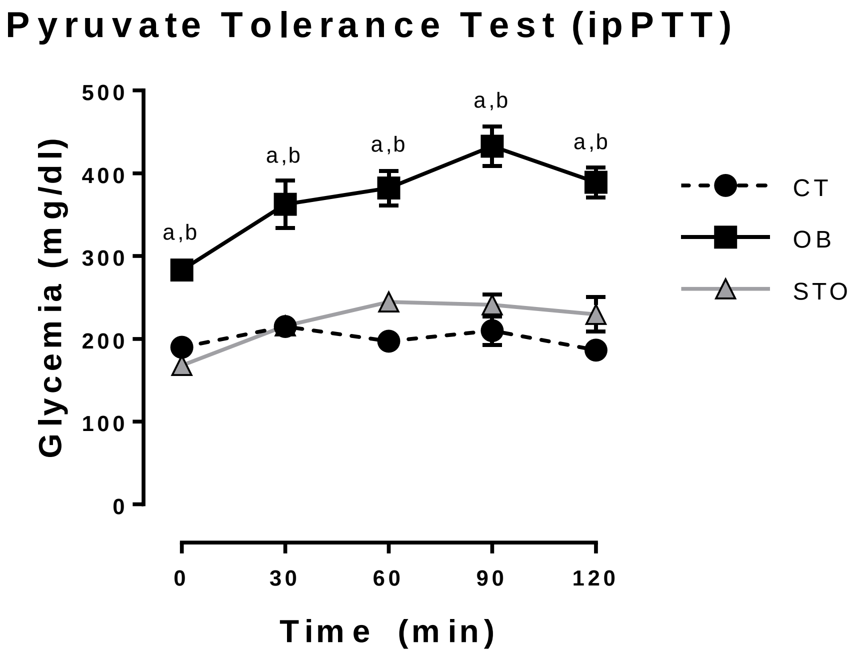

Bonferroni's multiple comparisons test	95.00% CI of diff.	Significant?	Summary	Adjusted P Value
0				
CT vs. OB	-145.8 to -41.24	Yes	***	0.0001
CT vs. STO	-30.26 to 74.26	No	ns	0.9176
OB vs. STO	63.24 to 167.8	Yes	****	<0.0001
30				
CT vs. OB	-199.8 to -95.24	Yes	****	<0.0001
CT vs. STO	-52.76 to 51.76	No	ns	>0.9999
OB vs. STO	94.74 to 199.3	Yes	****	<0.0001
60				
CT vs. OB	-237.6 to -133.1	Yes	****	<0.0001
CT vs. STO	-99.76 to 4.758	No	ns	0.0871
OB vs. STO	85.58 to 190.1	Yes	****	<0.0001
90				
CT vs. OB	-275.1 to -170.6	Yes	****	<0.0001
CT vs. STO	-83.26 to 21.26	No	ns	0.4514
OB vs. STO	139.6 to 244.1	Yes	****	<0.0001
120				
CT vs. OB	-254.9 to -150.4	Yes	****	<0.0001
CT vs. STO	-95.42 to 9.091	No	ns	0.1400
OB vs. STO	107.2 to 211.8	Yes	****	<0.0001

Figure 2B: AUC during ipPTT

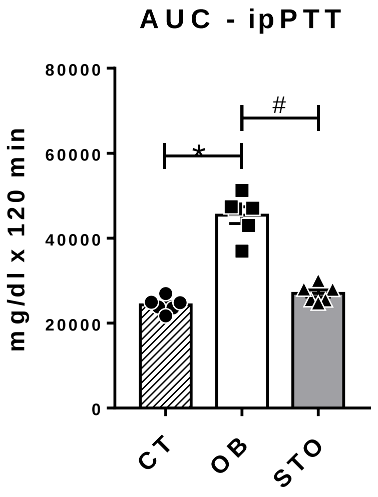

Group A	Group B	Group C
CT	OB	STO
Y	Y	Y
23820	36975	27975
23550	51210	30090
24825	43005	25515
21735	46875	25545
26985	47385	28050
24900	47040	24765

Bonferroni's multiple comparisons test	Mean Diff.	95.00% CI of diff.	Significant?	Summary	Adjusted P Value
CT vs. OB	-21113	-26119 to -16106	Yes	****	<0.0001
CT vs. STO	-2688	-7694 to 2319	No	ns	0.5061
OB vs. STO	18425	13419 to 23431	Yes	****	<0.0001

Figure 2C and D: Phospho-Akt^{S473} - CTL x OB

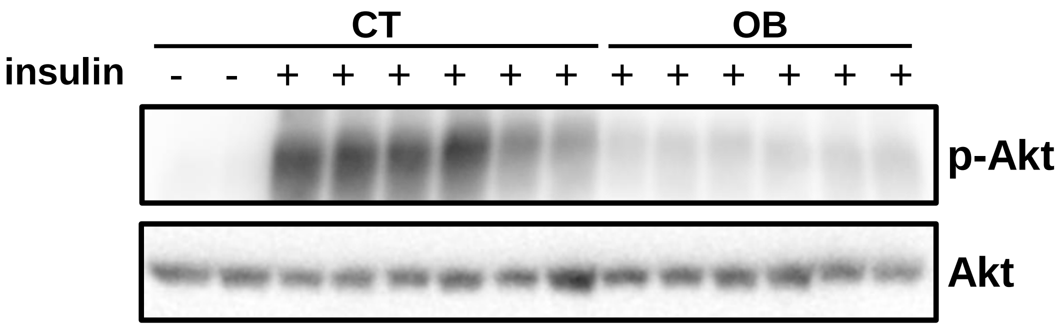

Quantification

CT	OB
Y	Y
137.90	19.79
125.68	18.91
115.21	18.65
109.95	14.57
75.59	16.52
35.68	22.81

Phospho-Akt^{ser473}

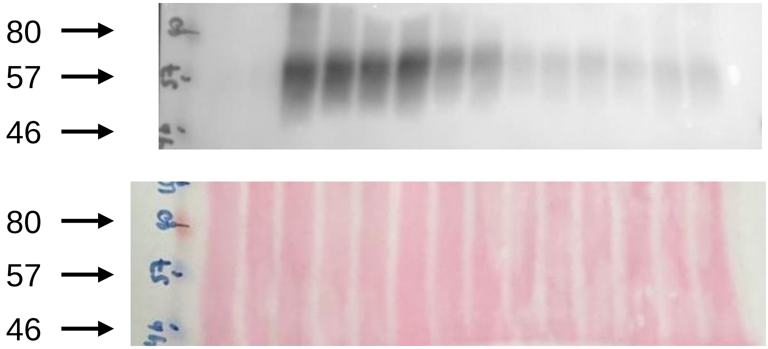

Total Akt

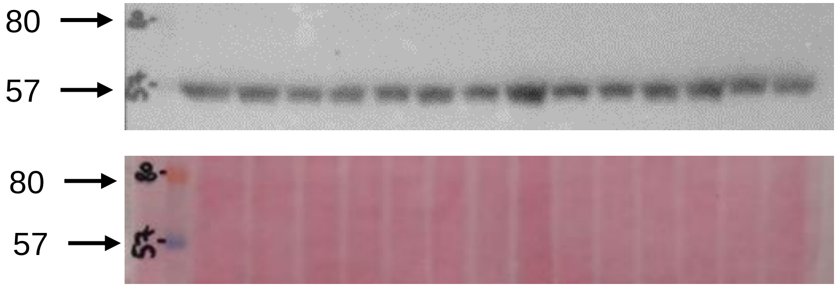

PRISM analysis

Column B	OB
vs.	vs.
Column A	CT
Unpaired t test	
P value	0.0004
P value summary	***
Significantly different (P < 0.05)?	Yes
One- or two-tailed P value?	Two-tailed
t, df	t=5.259 df=10

Figure 2C and E: Phospho-Akt^{S473} – OB x STO

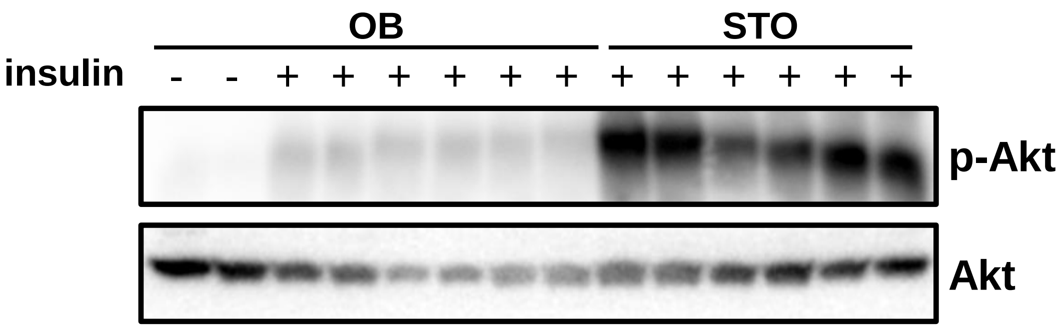

Phospho-Akt^{ser473}

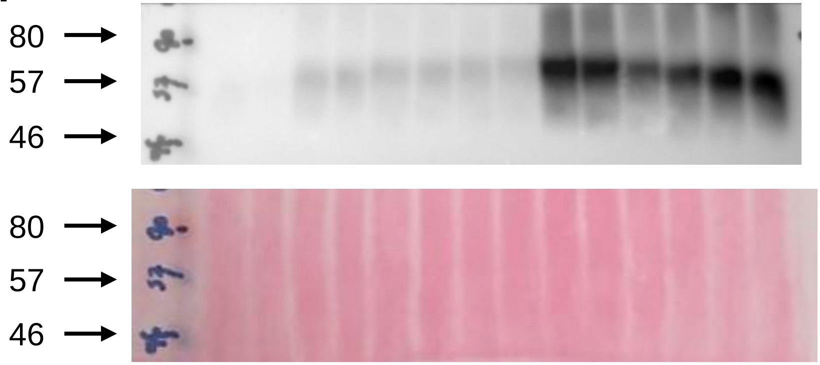

Total Akt

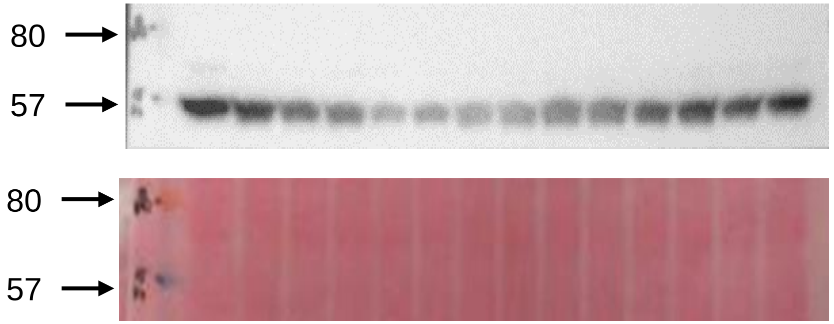

Quantification

OB	STO
Y	Y
51.14	624.63
65.18	580.10
127.05	353.33
128.39	340.56
107.37	483.11
120.87	396.66

PRISM analysis

Column B	STO
vs.	vs.
Column A	OB
Unpaired t test	
P value	<0.0001
P value summary	****
Significantly different (P < 0.05)?	Yes
One- or two-tailed P value?	Two-tailed
t, df	t=7.153 df=10

Figure 3A and B: PTP1B – CT x OB x STO

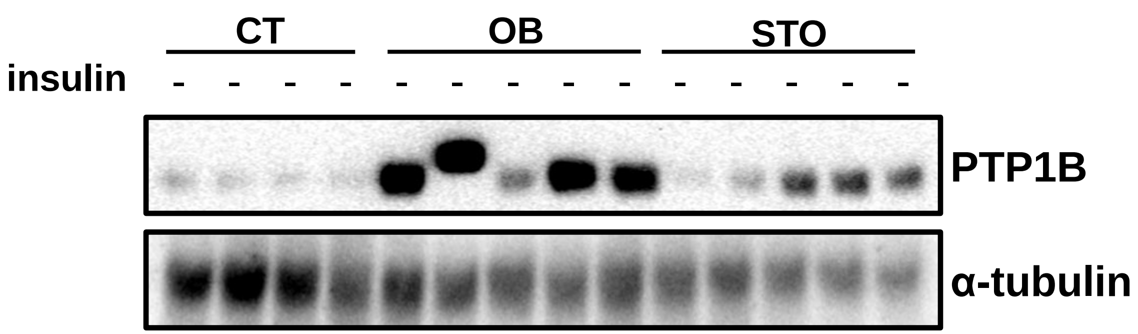

Quantification

CT -	OB -	STO -
138.67270	1221.56600	119.30930
74.71388	1545.35700	218.36850
67.43013	474.62010	725.17300
119.18330	1558.62100	842.73320
	1119.76200	780.90480

PTP1B

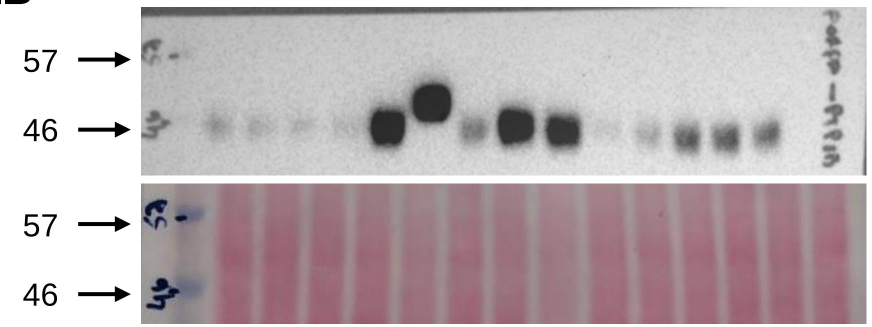

α-tubulin

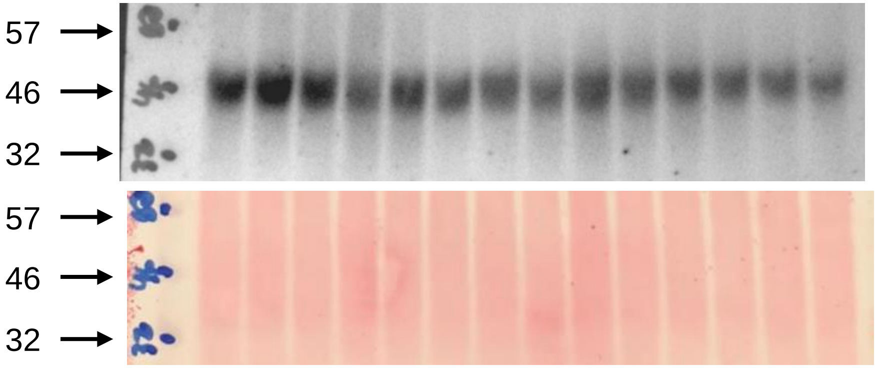

PRISM analysis

	Ordinary one-way ANOVA<br>Multiple comparisons						
1	Number of families	1					
2	Number of comparisons per family	3					
3	Alpha	0.05					
4							
5	**Bonferroni's multiple comparisons test**	**Mean Diff.**	**95.00% CI of diff.**	**Significant?**	**Summary**	**Adjusted P Value**	
6	CT - vs. OB -	-1084	-1721 to -446.9	Yes	**	0.0017	A-B
7	CT - vs. STO -	-437.3	-1074 to 199.8	No	ns	0.2371	A-C
8	OB - vs. STO -	646.7	46.03 to 1247	Yes	*	0.0340	B-C

Figure 3C and D: PTP1B – CT x OB

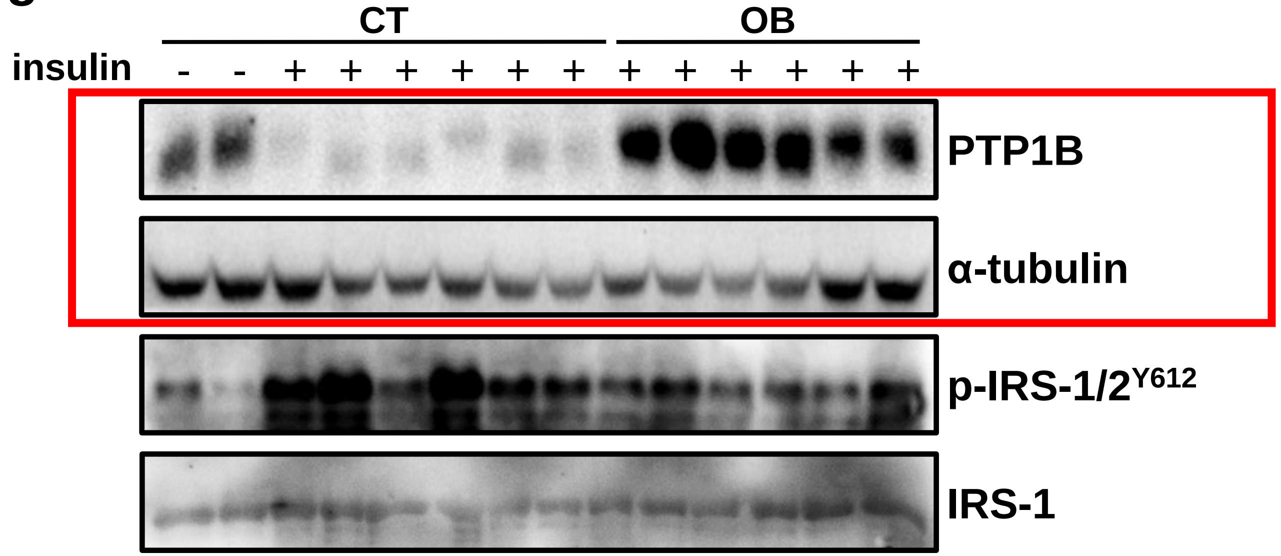

Quantification

Group A	Group B
CT	OB
Y	Y
52.11000	624.25000
87.62000	984.88000
79.30000	971.82000
71.37000	683.79000
158.90000	349.53000
150.70000	296.19000

PTP1B

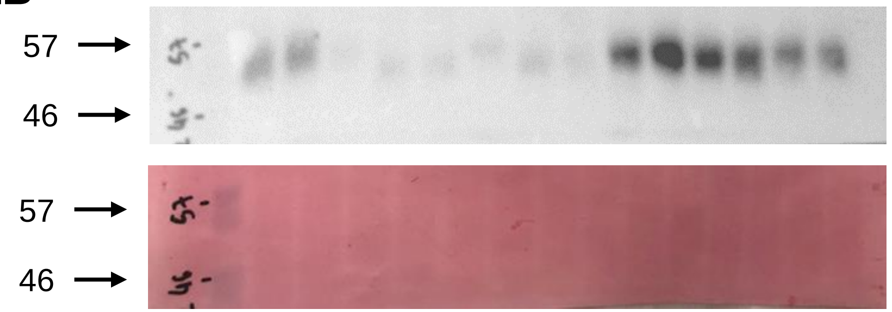

PRISM analysis

Unpaired t test	
Table Analyzed	ct PTP1B / a tubulin
Column B	OB
vs.	vs.
Column A	CT
Unpaired t test	
P value	0.0011
P value summary	**
Significantly different (P < 0.05)?	Yes
One- or two-tailed P value?	Two-tailed
t, df	t=4.542 df=10

α-tubulin

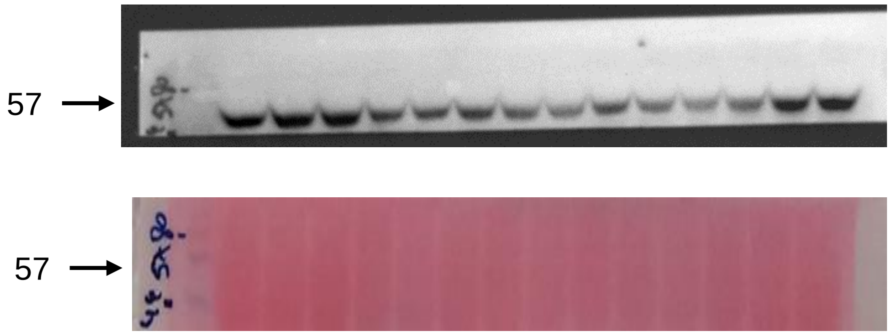

Figure 3E: Correlation PTP1B x p-Akt – CT x OB

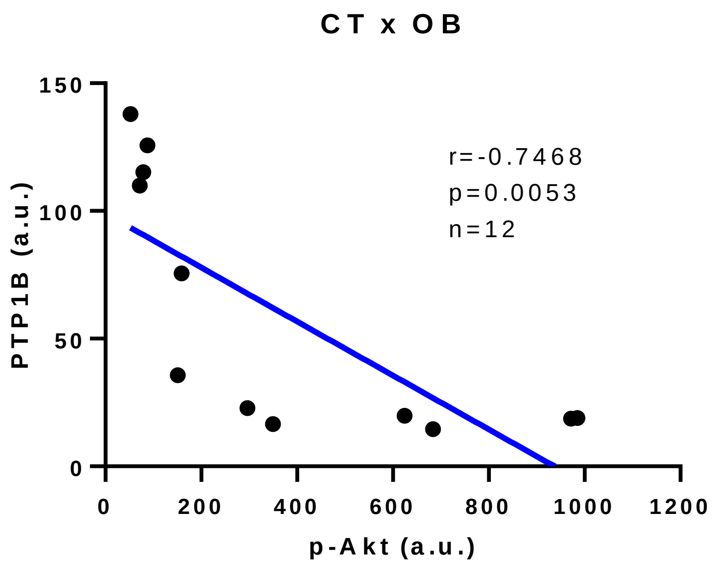

	Correlation	A<br>PTP1B vs. p-Akt
		Y
1	Pearson r	
2	r	-0.7468
3	95% confidence interval	-0.9245 to -0.3026
4	R squared	0.5577
5		
6	P value	
7	P (two-tailed)	0.0053
8	P value summary	**
9	Significant? (alpha = 0.05)	Yes
10		
11	Number of XY Pairs	12
12		

Figure 3C and F: p-IRS1/2^{Y612} – CT x OB

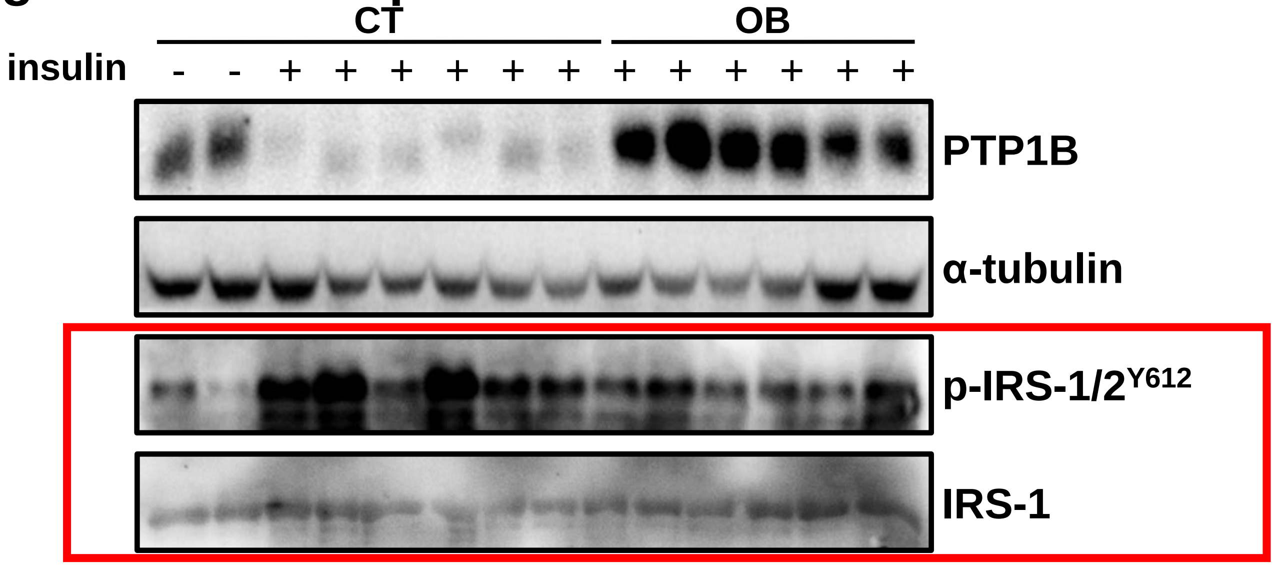

p-IRS1/2^{Y612}

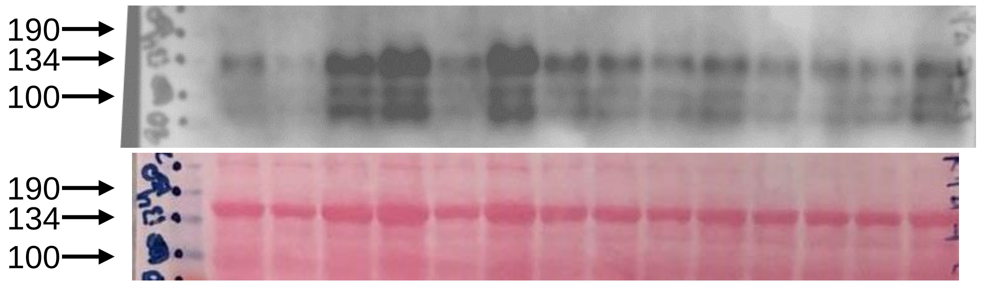

IRS1

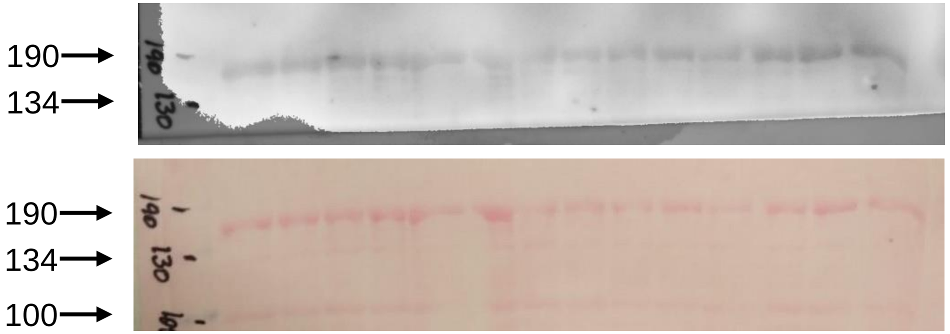

Quantification

CT	OB
Y	Y
55.87	39.51
72.27	45.51
89.75	24.38
187.08	24.23
107.65	16.55
87.38	32.21

PRISM analysis

Column B	OB
vs.	vs.
Column A	CT
Unpaired t test	
P value	0.0048
P value summary	**
Significantly different (P < 0.05)?	Yes
One- or two-tailed P value?	Two-tailed
t, df	t=3.602 df=10

Figure 3G and H: PTP1B – OB x STO

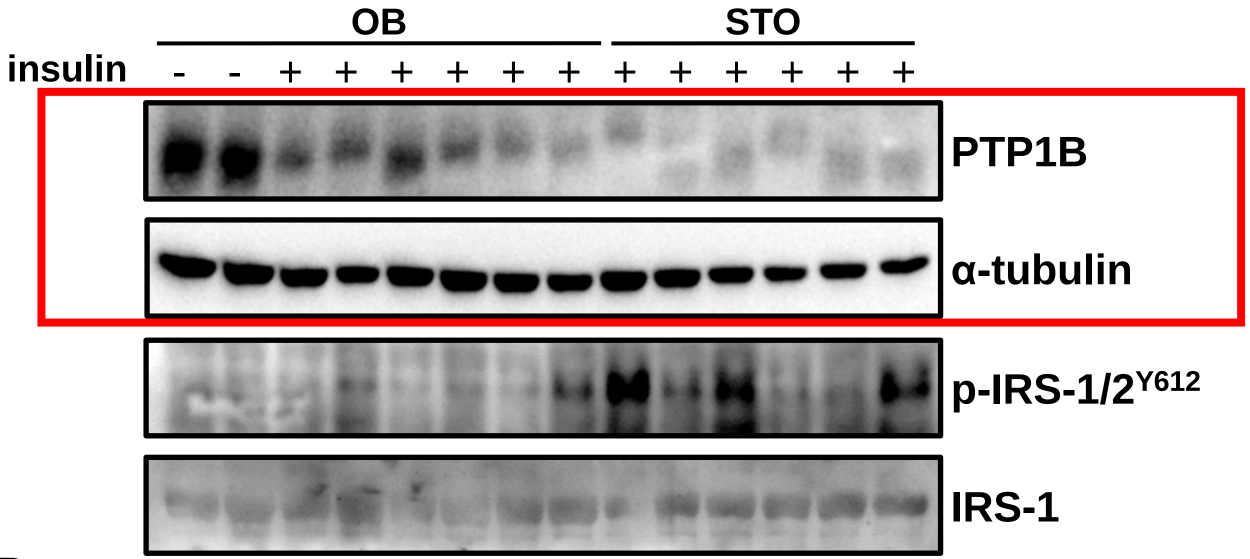

Quantification

Group A	Group B
OB	STO
Y	Y
118.93000	35.63000
138.70000	8.49000
143.05000	28.16000
92.12000	27.93000
65.72000	32.81000
41.48000	23.59000

PTP1B

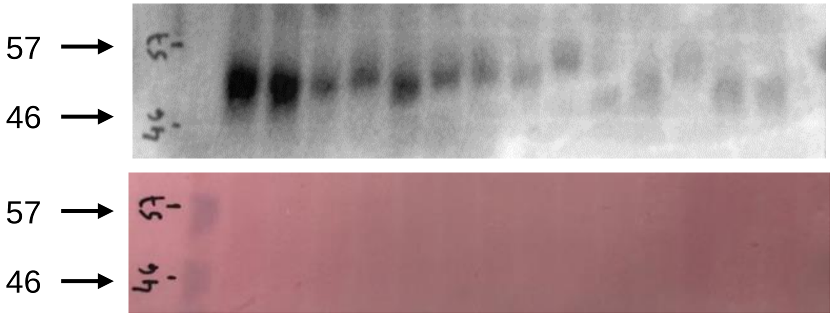

α-tubulin

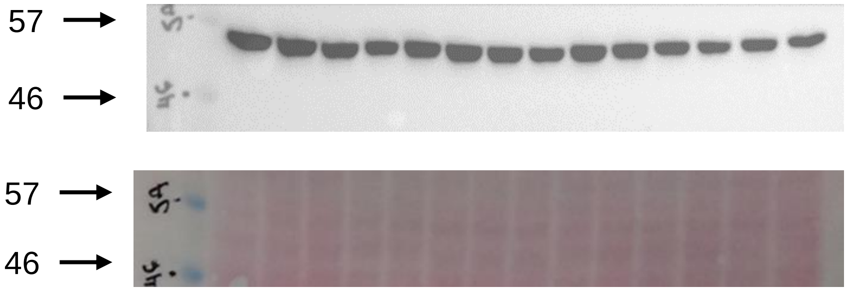

PRISM analysis

Unpaired t test	
Table Analyzed	$ PTP1B / a tubulin
Column B	STO
vs.	vs.
Column A	OB
Unpaired t test	
P value	0.0015
P value summary	**
Significantly different (P < 0.05)?	Yes
One- or two-tailed P value?	Two-tailed
t, df	t=4.307 df=10

Figure 3I: Correlation PTP1B x p-Akt – OB x STO

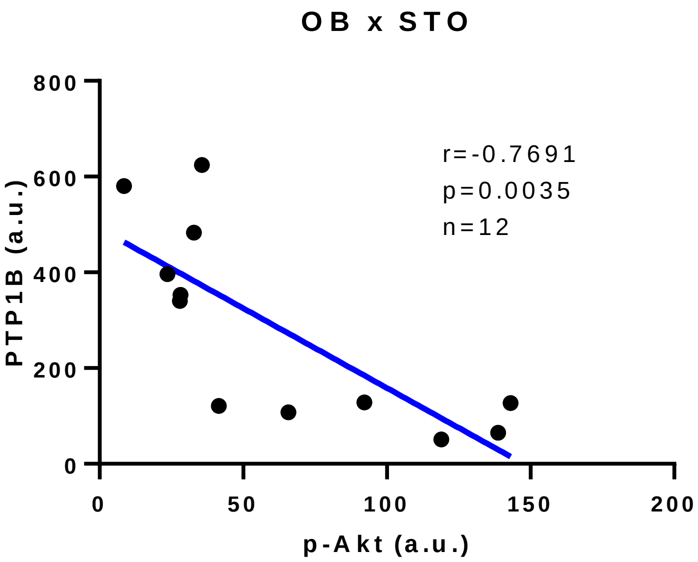

Correlation	PTP1B vs. p-Akt
	Y
Pearson r	
r	-0.7691
95% confidence interval	-0.9317 to -0.3494
R squared	0.5915
P value	
P (two-tailed)	0.0035
P value summary	**
Significant? (alpha = 0.05)	Yes
Number of XY Pairs	12

Figure 3G and J: p-IRS1/2^{Y612} – OB x STO

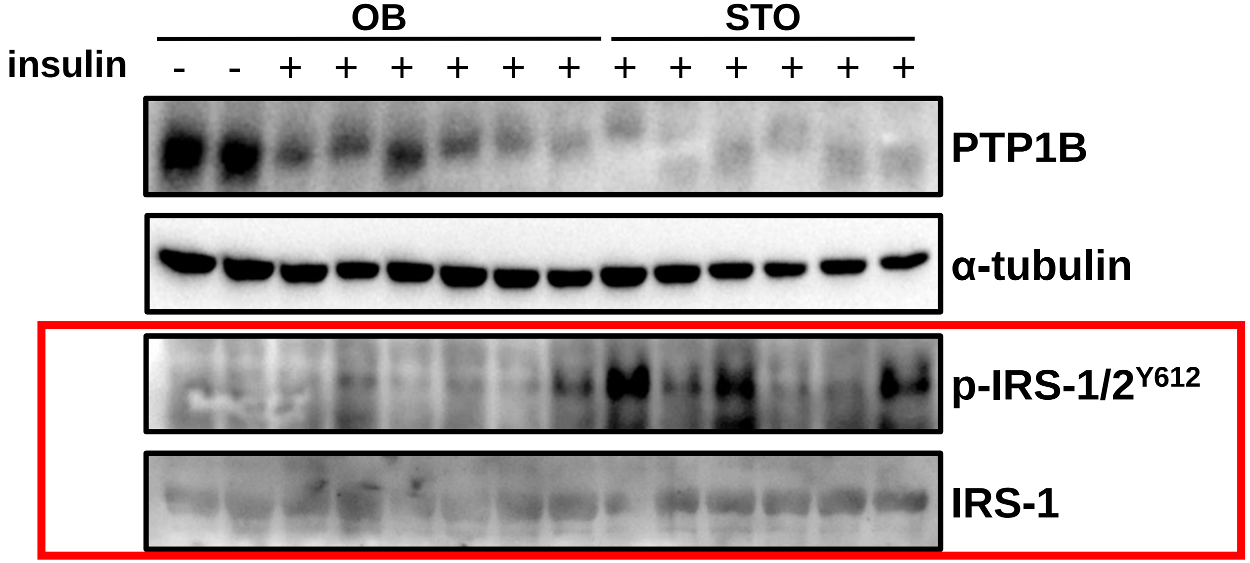

p-IRS1/2^{Y612}

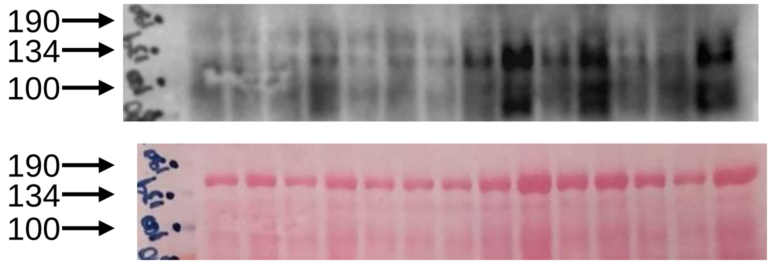

IRS1

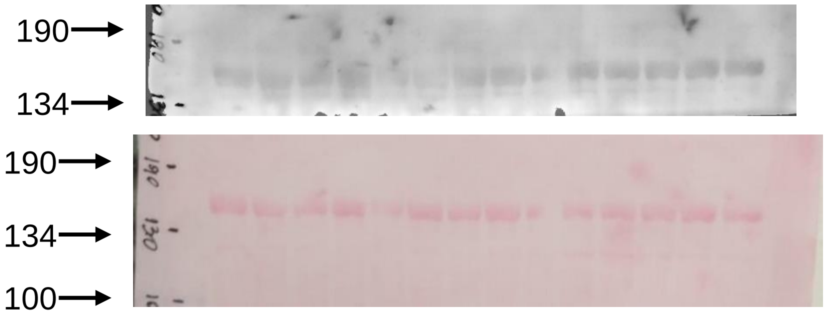

Quantification

OB	STO
Y	Y
45.69	506.21
96.55	191.64
60.37	278.58
136.69	149.80
67.02	154.55
193.68	254.02

PRISM analysis

Column B	STO
vs.	vs.
Column A	OB
Unpaired t test	
P value	0.0248
P value summary	*
Significantly different (P < 0.05)?	Yes
One- or two-tailed P value?	Two-tailed
t, df	t=2.639 df=10

Figure 4B: Correlation between Blood glucose and PTP-1B

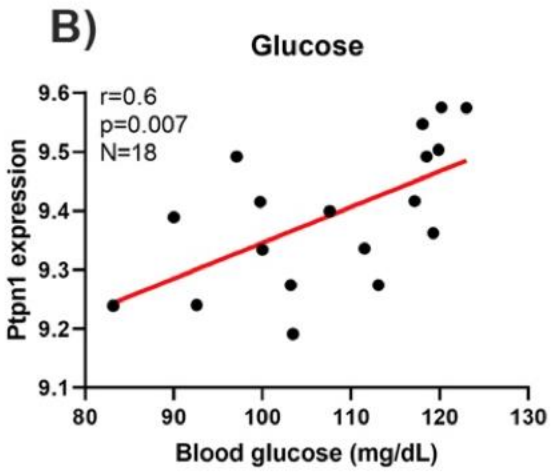

	Correlation	A<br>Blood glucose vs. Ptpn1
		Y
1	Pearson r	
2	r	0.6041
3	95% confidence interval	0.1911 to 0.8353
4	R squared	0.3649
5		
6	P value	
7	P (two-tailed)	0.0079
8	P value summary	**
9	Significant? (alpha = 0.05)	Yes
10		
11	Number of XY Pairs	18
12		

Figure 4C: Correlation between Locomotor activity and PTP-1B

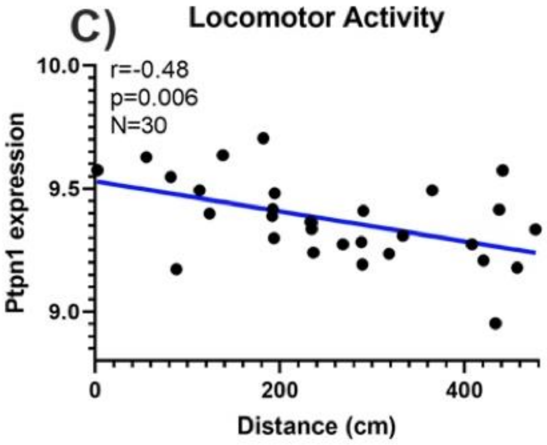

	Correlation	A<br>Locomotor activity vs. Ptpn1
		Y
1	Pearson r	
2	r	-0.4854
3	95% confidence interval	-0.7198 to -0.1516
4	R squared	0.2356
5		
6	P value	
7	P (two-tailed)	0.0066
8	P value summary	**
9	Significant? (alpha = 0.05)	Yes
10		
11	Number of XY Pairs	30

Figure 4D: Correlation between Liver mass and PTP-1B

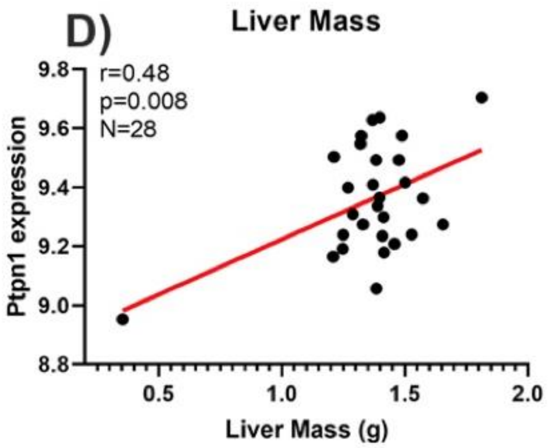

	Correlation	A<br>Liver Mass<br>vs.<br>Ptpn1
		Y
1	Pearson r	
2	r	0.4871
3	95% confidence interval	0.1393 to 0.7279
4	R squared	0.2372
5		
6	P value	
7	P (two-tailed)	0.0086
8	P value summary	**
9	Significant? (alpha = 0.05)	Yes
10		
11	Number of XY Pairs	28

Figure 4E: Correlation between Body weight and PTP-1B

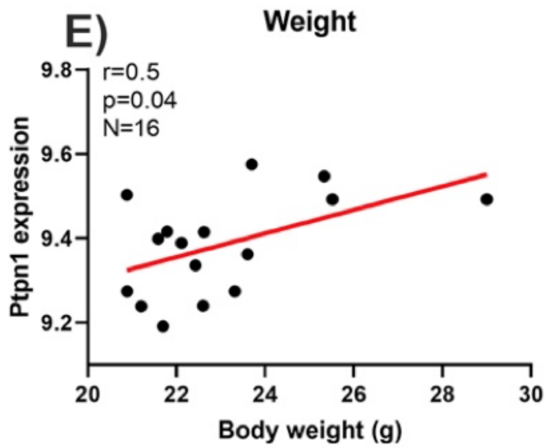

	Correlation	A<br>Body weight<br>vs.<br>Ptpn1
		Y
1	Pearson r	
2	r	0.5025
3	95% confidence interval	0.00905 to 0.7991
4	R squared	0.2525
5		
6	P value	
7	P (two-tailed)	0.0473
8	P value summary	*
9	Significant? (alpha = 0.05)	Yes
10		
11	Number of XY Pairs	16

Pipeline graph to describing the steps of bioinformatic analysis

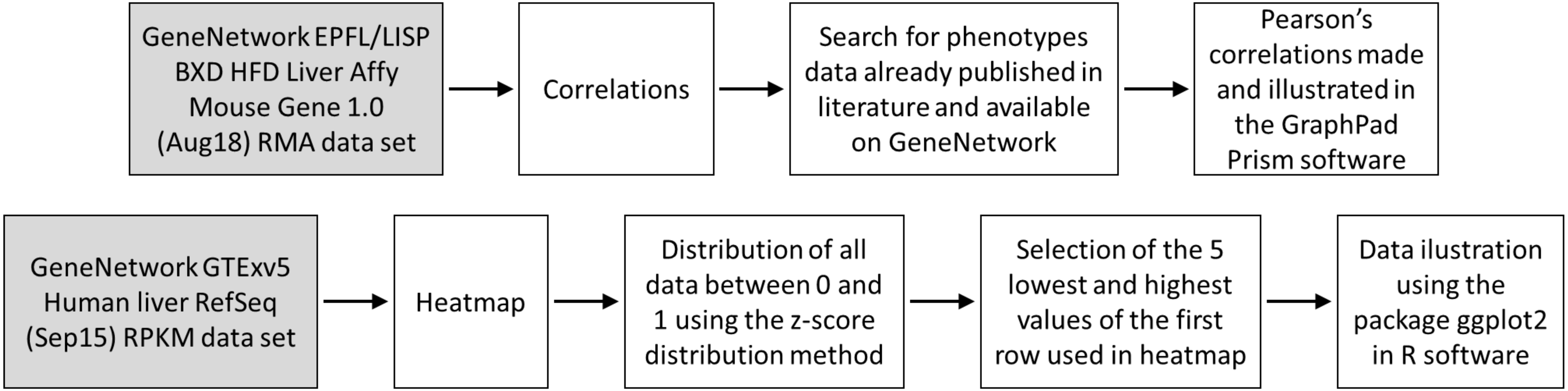

Supplement: Supplementary file 1 [file ijms-22-06402-s001.zip › ijms-1108247-supplementary.pdf]
